# Advancing and receding contact angle investigations for highly sticky and slippery aluminum surfaces fabricated from nanostructured anodic oxide†

**DOI:** 10.1039/c8ra07712f

**Published:** 2018-11-06

**Authors:** Daiki Nakajima, Tatsuya Kikuchi, Shungo Natsui, Ryosuke O. Suzuki

**Affiliations:** Faculty of Engineering, Hokkaido University N13-W8, Kita-ku Sapporo Hokkaido 060-8628 Japan kiku@eng.hokudai.ac.jp

## Abstract

The fabrication of sticky and slippery superhydrophobic aluminum was achieved by anodizing in pyrophosphoric acid and modification with self-assembled monolayers (SAMs). In addition, the corresponding sliding behaviors of a water droplet were investigated by contact angle measurements and direct observations. For the formation of anodic alumina nanofibers, 4N aluminum plates were anodized in a concentrated pyrophosphoric acid solution at 25–75 V. The morphology of the anodic oxide successively changed to barrier oxide, porous oxide, nanofibers, bundle structures with many nanofibers, and then weak nanofibers during anodizing. The anodized specimens were immersed in a fluorinated phosphonic acid/ethanol solution to form SAMs on the surface of the anodic oxide. The contact angle hysteresis drastically changed with anodizing time: it increased with the formation of porous oxide, decreased for the nanofibers and bundle structures, and then increased once again for the weak nanofibers. Correspondingly, the adhesion interaction between the water droplet and the aluminum surface also drastically changed to show sticky, slippery, and sticky behaviors with anodizing time. More sticky and slippery aluminum surfaces can be obtained by anodizing at higher voltages. The slippery behavior was further improved through two distinct anodizing processes with the formation of ordered alumina nanofibers. A superhydrophobic aluminum surface with coexisting sticky and slippery properties was fabricated by the selective anodizing method.

## Abstract 2

Highly sticky and slippery superhydrophobic aluminum surfaces were successfully fabricated by pyrophosphoric acid anodizing.

## Graphical abstract

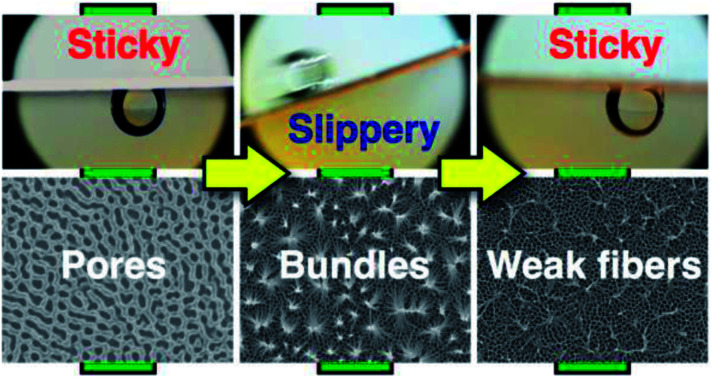

## Introduction

Anodizing (anodization) is one of the most important surface finishing processes for valve metals, such as aluminum, titanium, magnesium, and their alloys. In particular, aluminum is often anodized for mechanical property improvement,^1–4^ corrosion protection,^5–9^ electronic applications,^10,11^ and nanostructural engineering.^12–14^ The morphology of anodic aluminum oxide is determined by the electrolyte solution used during anodizing.^15–20^ Anodizing aluminum in a pyrophosphoric acid (H_4_P_2_O_7_) solution causes the formation of numerous nanoscale anodic alumina nanofibers.^21,22^ Although similar alumina nanofibers were formed by long-term anodizing in typical electrolyte solutions, such as oxalic and phosphoric acids, followed by chemical dissolution of the anodic oxide,^23–25^ pyrophosphoric acid anodizing (PyAA) has the following major advantages: (a) the rapid formation of alumina nanofibers within 2 min under the appropriate operating conditions;^22^ (b) the ability to precisely control the surface nanostructures, such as independent standing nanofiber arrays, bundle structures with different scales, and high-aspect-ratio bending nanofibers;^26^ and (c) the successful fabrication of highly ordered alumina nanofibers *via* two distinct anodizing processes.^27^ These fibrous oxides can also be fabricated on various commercially available aluminum alloys, such as 1N30, 3004, and 8021.^28^

PyAA allows control of the water wettability over a wide range of values on the aluminum surface. A nanofiber-covered aluminum surface exhibits superhydrophilic properties with a water contact angle (WCA) of less than 10° observed within 0.1 seconds after placement of the water drop.^29,30^ Rapid drying and snow-sliding behaviors were observed on these aluminum surfaces covered with alumina nanofibers. Conversely, the WCA drastically changes to show superhydrophobic behavior and is over 150° when the surface of the alumina nanofibers is modified with a self-assembled monolayer (SAM).^26^ This superhydrophobic aluminum surface shows high light reflectance, measuring more than 99% over the whole visible spectrum (compared with an electropolished aluminum surface).

Although all superhydrophobic surfaces possess similar static WCAs that are greater than 150°, the adhesion interaction between the water droplet and the superhydrophobic surface may vary according to the surface morphology. For example, a lotus leaf exhibits superhydrophobic behavior, as observed by the WCA measurement, and the water droplet can easily slide from the surface (*i.e.*, “slippery” surface).^31,32^ By contrast, while a rose petal also exhibits similar superhydrophobic behavior with respect to the contact angle, the water droplet strongly adsorbs on its surface (*i.e.*, “sticky” surface).^33^ Because both slippery and sticky surfaces are of considerable interest for various industrial applications, such as self-cleaning surfaces,^34,35^ heat-exchange devices,^36–38^ single-molecule spectroscopy,^39,40^ and micromanipulators,^41^ the fabrication of slippery and sticky aluminum surfaces would expand the applicability of aluminum and its alloys.

Herein, we demonstrate the fabrication of slippery and sticky aluminum surfaces covered with anodic alumina nanofibers *via* PyAA under various electrochemical conditions and subsequent modification with fluorinated SAMs. The effects of the morphological properties of the alumina nanofibers, such as the length, density, and periodicity, on the adhesion interaction between the water droplet and the surface were investigated by WCA measurements. The adhesion interaction changed drastically with anodizing time and the consequent morphology. We successfully fabricated a highly slippery aluminum surface with a sliding angle of less than 2° and a highly sticky aluminum surface with a non-sliding angle of 180° by controlling the surface morphology *via* PyAA. Moreover, we also demonstrate a superhydrophobic aluminum surface with coexisting sticky and slippery properties that was fabricated *via* the selective PyAA method.

## Experimental

### Pretreatment of aluminum specimens

Commercially available aluminum plates (purity: 99.99 wt%, size: 20 mm × 40 mm, thickness: 400 μm, Nippon Light Metal, Japan) were used as the starting aluminum specimens. The aluminum plates were immersed in ethanol and then ultrasonically degreased for 10 min. The bottom half of the specimens (*i.e.*, 20 mm × 20 mm area) was electrochemically polished in a 13.6 M acetic acid/2.56 M perchloric acid solution (*T* < 280 K) at 28 V for 1 min. A high-purity aluminum plate was used as the cathode, and the electropolishing solution was stirred by a magnetic stirrer bar.

### Anodizing of aluminum

A commercially available concentrated pyrophosphoric acid solution (concentration: 74.0 wt%, Kanto Chemical, Japan) was used as the anodizing electrolyte solution. An electropolished aluminum specimen was immersed in the pyrophosphoric acid solution (solution volume: 100 cm^3^, inner diameter of the glass cell: 55 mm, *T* = 293 K). A platinum plate (purity: 99.95 wt%, size: 16 mm × 28 mm, thickness: 100 μm, Furuya Metal, Japan) was used as the cathode. The aluminum anode and platinum cathode were set parallel at a distance of 20 mm in the electrochemical cell. Then, the aluminum specimen was anodized at 25–75 V for 60 min to form anodic alumina nanofibers (constant voltage PyAA). The anodizing solution was stirred by a magnetic stirrer bar at 1.0 s^−1^ during PyAA and maintained at a constant temperature using a water bath (UCT-1000, AS ONE, Japan). A direct current power supply (PWR-400H/400M, Kikusui, Japan) was used for PyAA. For comparison, typical porous alumina was also formed by anodizing in a 0.3 M oxalic acid solution at 293 K and 50 V for 60 min (oxalic acid anodizing: OAA).

An ordered anodic alumina nanofiber array was fabricated by two distinct anodizing processes.^27^ First, the electropolished aluminum specimens were anodized in a 0.3 M oxalic acid solution at 293 K and 50 V for 2 h to form ordered porous alumina (OAA). The porous alumina was dissolved in a 0.2 M chromic acid/0.51 M phosphoric acid solution (*T* = 353 K) for 20 min, forming an ordered dimple array on the aluminum surface. After chemical dissolution, the specimens were anodized again in pyrophosphoric acid solution for 60 min to form an ordered alumina nanofiber array (PyAA).

### Modification of SAMs

After anodizing, the surface of the anodic oxide was modified with 3,3,4,4,5,5,6,6,7,7,8,8,8-tridecafluorooctylphosphonic acid (FOPA). In this modification, (a) the anodized specimens were placed into a vial filled with 0.5 mM FOPA/ethanol solution (volume: 30 mL), (b) the vial was sealed with a screw cap, and (c) the vial was placed in an incubator (Heratherm IMC18, Thermo Fisher Scientific, USA) at 313 K for 24 h. After SAM modification, the specimens were stored in a desiccator after washing with ethanol.

### Characterization

The anodized specimens were observed by field-emission scanning electron microscopy (FE-SEM, JSM-6500F, JEOL, Japan). A platinum electroconductive layer was sputter-coated on the surface of the specimens before SEM observation (MSP-1S, Vacuum Device, Japan).

The WCAs on the anodized specimens were examined by an optical contact angle meter at room temperature (DM-501, Kyowa Interface Science, Japan).^42,43^ Although high-precision WCA measurements such as high-precision drop shape analysis were already reported,^44–46^ standard advancing and receding contact angle measurements were used for the fabrication of sticky and slippery aluminum surfaces in the present investigation. Ultra pure water droplets with a resistance of 18.2 MΩ cm were formed on the surface using an auto dispenser and a microsyringe with a flow rate of 2 μL s^−1^, and the advancing contact angle (ACA) and receding contact angle (RCA) were recorded by a CCD camera. Here, the ACA was defined as the angle measured when the droplet had the maximum volume for the same interface area during water injection. Conversely, the RCA was defined as the angle measured when the droplet had the minimum volume for the same interface area during water suction. The RCA value was defined as zero when the triple point (three-phase contact point) was fixed during water suction. The ACA and RCA values of the water droplets were fitted using a circle fitting method. WCA measurements were performed at five different positions on each specimen, and then, the values were averaged without including the maximum and minimum values.

The slipping behavior of the water droplet formed on the anodized specimens was observed using a rotating stage with a stage controller (SKIDS-60YAW and Mark-102, Sigmakoki, Japan) and a digital microscope (Hidemicron Pro2, Tec, Japan). A water droplet (20 μL) was placed on the horizontal anodized specimen, and then, the specimen was rotated counterclockwise at a rate of 5° s^−1^ to cause the droplet to slide.

## Results and discussion

**Fig. 1:**
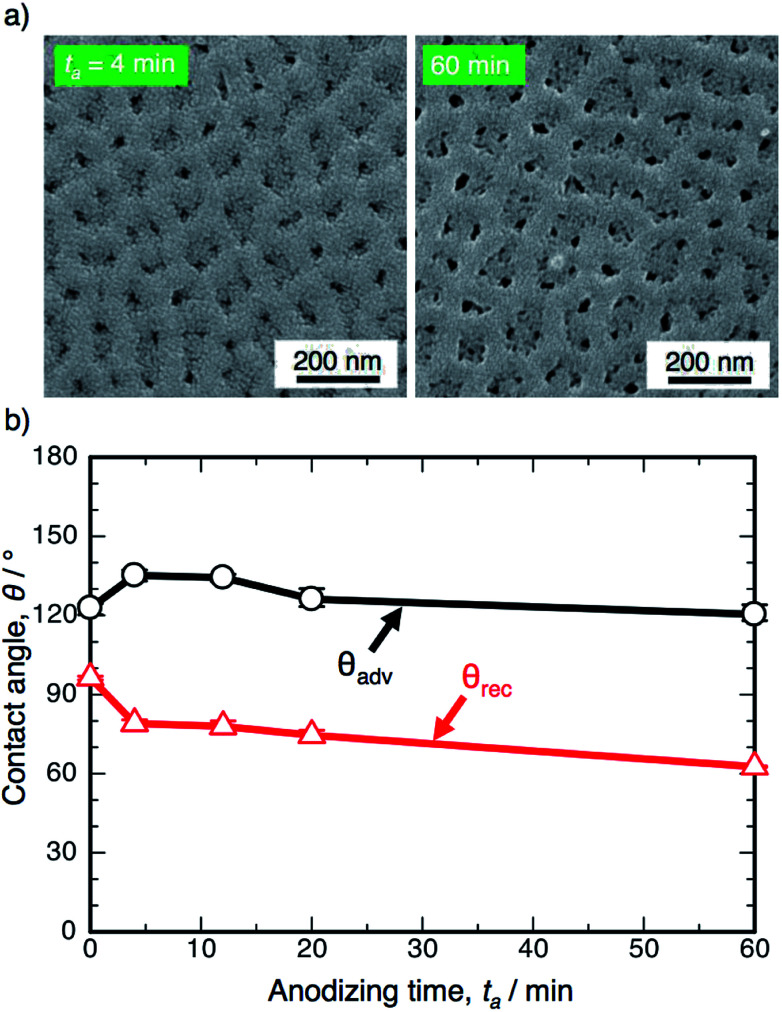
(a) SEM images of the surface of the specimen anodized in 0.3 M oxalic acid solution at 293 K and 50 V for 4 min and 60 min. (b) Changes in the advancing contact angle, *θ*_adv_, and receding contact angle, *θ*_rec_, on the anodized specimen with anodizing time, *t*_a_.

Fig. 1a shows the SEM images of the surface formed by OAA in a 0.3 M oxalic acid solution at 293 K and 50 V for *t*_a_ = 4 min and 60 min. Many nanosize pores with diameters of approximately 15–35 nm were observed over the entire surface, and typical porous alumina was obtained in the initial stage of OAA. The morphology of the porous alumina was nearly unchanged by the additional anodizing process carried out for 60 min, even though the diameter of the pores increased slightly due to chemical dissolution in oxalic acid. The electropolished and anodized aluminum specimens were immersed in a 0.5 mM FOPA/ethanol solution to form SAMs on their surfaces, and the WCAs were measured using an optical contact angle meter.

Fig. 1b shows the changes in the ACA, *θ*_adv_, and RCA, *θ*_rec_, on the SAM-modified aluminum specimens with the anodizing time, *t*_a_. The electropolished aluminum surface exhibits weak hydrophobicity with *θ*_adv_ = 122.8°, since FOPA molecules consisting of trifluoromethyl groups (–CF_3_) self-assembled on the aluminum oxide upon immersion in FOPA/ethanol solution.^47–50^ The *θ*_adv_ of the anodized specimens slightly increased to approximately 135° in the initial stage and then gradually decreased with increasing anodizing time. However, the *θ*_adv_ remained hydrophobic over the entire anodizing time. The *θ*_rec_ of the electropolished specimen was measured to be 96.4° and then decreased gradually with increasing anodizing time. Importantly, the difference between *θ*_adv_ and *θ*_rec_ corresponds to the spreading of the water droplet on the surface (*i.e.*, contact angle hysteresis, *θ*_adv_ − *θ*_rec_).^51–53^ The hysteresis of the electropolished surface was calculated to be 26.4°, and low spreading behavior was observed. The hysteresis increased to approximately 60° by OAA for each operating time, and the surfaces covered with porous alumina exhibited weak slippery behavior.

**Fig. 2:**
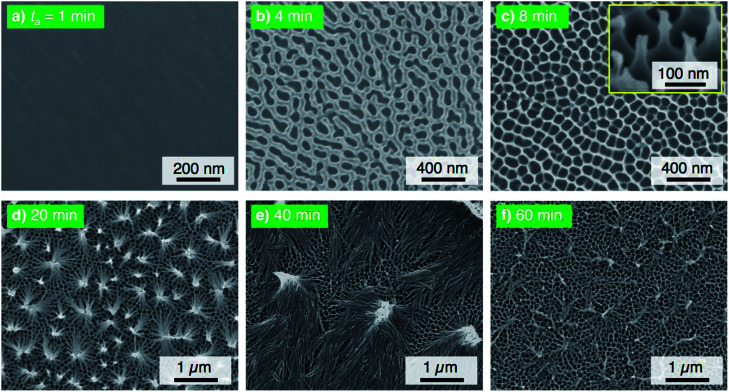
SEM images of the surface of the specimen anodized in concentrated pyrophosphoric acid solution at 293 K and 50 V for (a) 1 min through (f) 60 min.

The electropolished aluminum specimens were anodized in a concentrated pyrophosphoric acid solution under the same operating conditions at 293 K and 50 V. Fig. 2 shows the SEM images of the surface formed by PyAA for (a) *t*_a_ = 1 min through (f) 60 min. A thin, flat barrier anodic oxide was formed on the surface by PyAA for 1 min (Fig. 2a). As the anodizing time increased to 4 min, porous oxide with numerous nanopores was formed on the surface (Fig. 2b). The diameter of the pores formed by PyAA was larger than that of the pores formed by OAA (Fig. 1a) due to rapid chemical dissolution into the concentrated pyrophosphoric acid solution, and several continu ous grooves were also observed. The alumina cell was located around the pores dissolved by further PyAA for 8 min, and porous oxide with narrow polygonal walls was obtained on the surface (Fig. 2c). In addition, the tiled image shows that nanoscale alumina fibers were formed at the apices of each network structure (inset of Fig. 2c). These alumina nanofibers grow *via* further PyAA and bend due to their flexibility. As a result, pyramidal bundle structures consisting of several tens of alumina nanofibers were fabricated on the surface (Fig. 2d). These nanofibers were composed of amorphous aluminum oxide without an electrolyte anion. The average size of the alumina bundles increased with anodizing time due to the formation of longer alumina nanofibers, and large bundle structures consisting of several hundreds of alumina nanofibers were fabricated (Fig. 2e). However, excess PyAA led to chemical dissolution of the pure alumina nanofibers into the pyrophosphoric acid solution, and a weak nanofiber structure was observed after PyAA for 60 min (Fig. 2f). In sum, the anodic nanostructures formed by PyAA changed into barrier oxide, porous oxide, alumina nanofibers, bundle structures with many alumina nanofibers, and weak nanofibers. These anodized specimens were also modified with FOPA-SAMs, and then, their WCAs were measured.

**Fig. 3:**
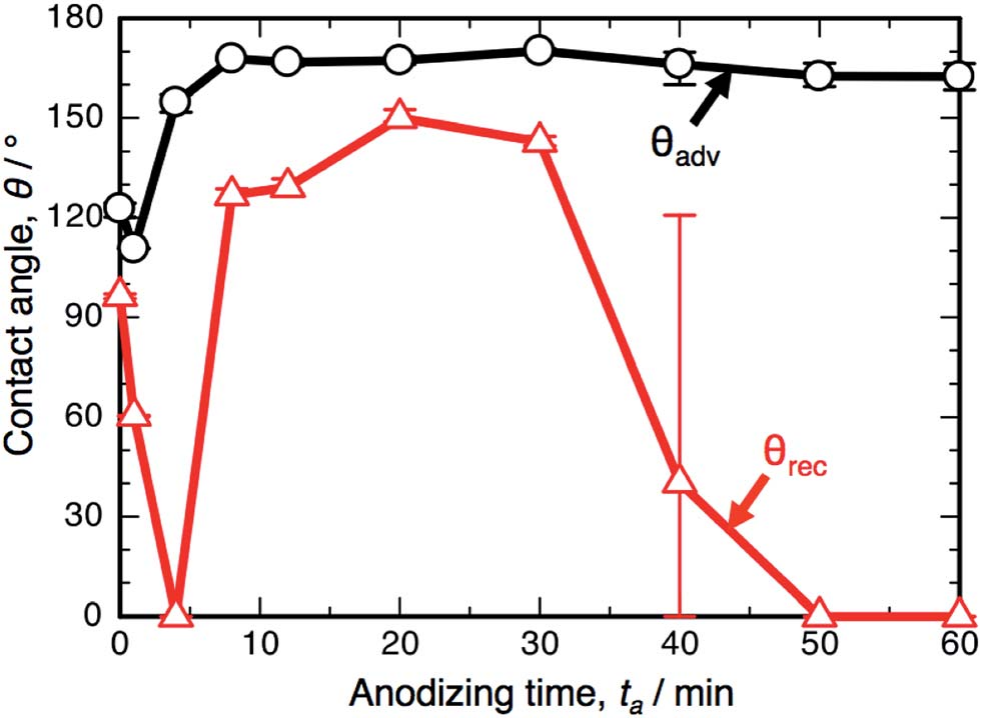
Changes in the advancing contact angle, *θ*_adv_, and receding contact angle, *θ*_rec_, on the anodized specimen with anodizing time, *t*_a_. The specimens were anodized in concentrated pyrophosphoric acid solution at 293 K and 50 V.

Fig. 3 shows the changes in *θ*_adv_ and *θ*_rec_ on the SAM-modified aluminum specimens with *t*_a_. The shape of the *θ*_adv_ curve was relatively simple: *θ*_adv_ rapidly increased to more than 150° after an initial slight decrease, and high *θ*_adv_ values of 162–170° were maintained upon further PyAA for up to 60 min. Thus, superhydrophobic aluminum surfaces with contact angles over 150° were successfully fabricated by PyAA after 4 min. On the other hand, *θ*_rec_ drastically changed with anodizing time. First, *θ*_rec_ decreased rapidly during the initial anodizing stage, and the lowest *θ*_rec_ value was obtained after anodizing for 4 min. Then, *θ*_rec_ rapidly increased to over 120° with further PyAA, and the highest *θ*_rec_ of 150° was obtained after PyAA for 20 min. Finally, *θ*_rec_ gradually decreased with anodizing time, and the lowest *θ*_rec_ values were measured once again after PyAA for more than 50 min. As a result, the contact angle hysteresis (*θ*_adv_ − *θ*_rec_) varied strongly with anodizing time and is divided into the following two regions: high hysteresis values of 154–162° were obtained with PyAA times of 4 min and over 50 min, and low hysteresis values of 17–40° were obtained with PyAA times of 8–30 min. Compared to the WCA measurements obtained by OAA (Fig. 1b), more slippery (low hysteresis) or sticky (high hysteresis) superhydrophobic aluminum surfaces can be obtained by PyAA for an appropriate operating time. Such slippery and sticky surfaces strongly depend on the morphology of the anodic oxide formed by PyAA. Therefore, further WCA measurements were performed on the aluminum specimens formed by various anodizing voltages.

**Fig. 4:**
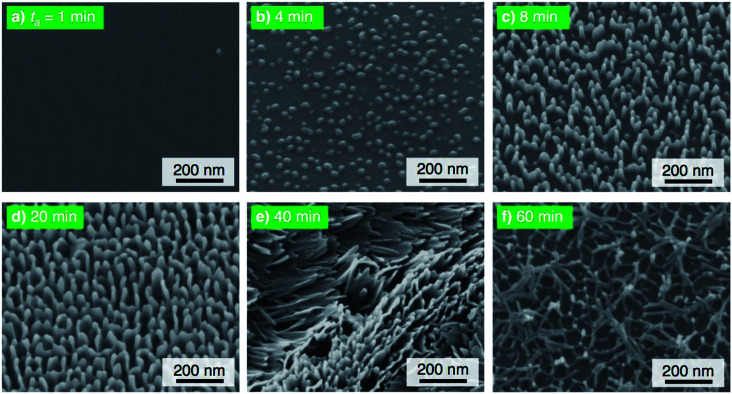
SEM images of the surface of the specimen anodized in concentrated pyrophosphoric acid solution at 293 K and 25 V for (a) 1 min through (f) 60 min.

The electropolished aluminum specimens were anodized at a relatively low voltage of 25 V in pyrophosphoric acid solution (293 K) for (a) *t*_a_ = 1 min through (f) 60 min, and the corresponding SEM images of the 20° tiled surface of the specimen are shown in Fig. 4. High-density alumina nanofibers gradually grew on the surface with increasing anodizing time up to 20 min (Fig. 4a–d) because the number of growth nuclei of the alumina nanofibers increases with decreasing anodizing voltage due to the formation of a smaller honeycomb structure at the bottom of the nanofibers, showing behavior that is similar to that observed in the formation of porous alumina.^54,55^ Then, linear bundle structures consisting of numerous alumina nanofibers were fabricated by further PyAA for 40 min (Fig. 4e). Finally, a weak nanofiber structure was observed upon excess PyAA for 60 min due to chemical dissolution (Fig. 4f). Compared to the growth behavior of the alumina nanofibers at 50 V (Fig. 2), the following major differences can be observed by PyAA at low voltage: (a) slow growth rate, (b) high density, and (c) formation of linear bundles.

**Fig. 5:**
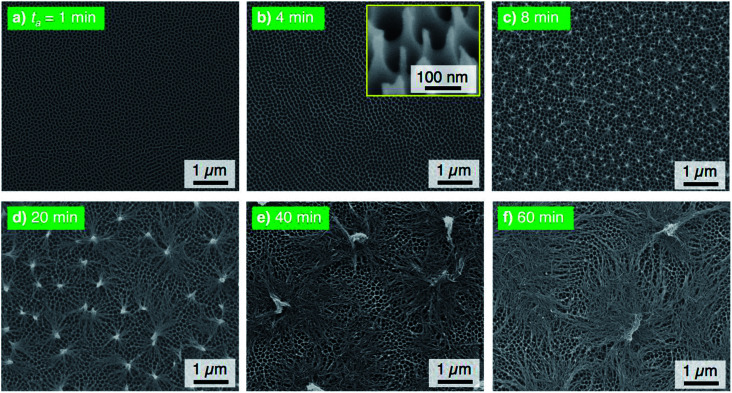
SEM images of the surface of the specimen anodized in concentrated pyrophosphoric acid solution at 293 K and 75 V for (a) 1 min through (f) 60 min.

The electropolished specimens were also anodized at a relatively high voltage of 75 V, and the surface morphologies of the anodized specimens are shown in Fig. 5. Because the anodic current density increased with the applied voltage, porous oxide, nanofibers, and pyramidal bundle structures grew rapidly on the surface with PyAA for 1–8 min (Fig. 5a–c). In addition, the density of the alumina nanofibers was lower than that obtained at the lower anodizing voltages. Because longer alumina nanofibers were formed by long-term PyAA, the pyramidal bundles became larger due to the absorption of surrounding alumina nanofibers (Fig. 5d and e). Finally, the aluminum surface was covered with longer alumina nanofibers after PyAA for 60 min (Fig. 5f). Compared to the growth behavior at 50 V, the following major differences can be observed by PyAA at high voltage: (a) high growth rate, (b) low density, and (c) formation of large bundle structures.

**Fig. 6:**
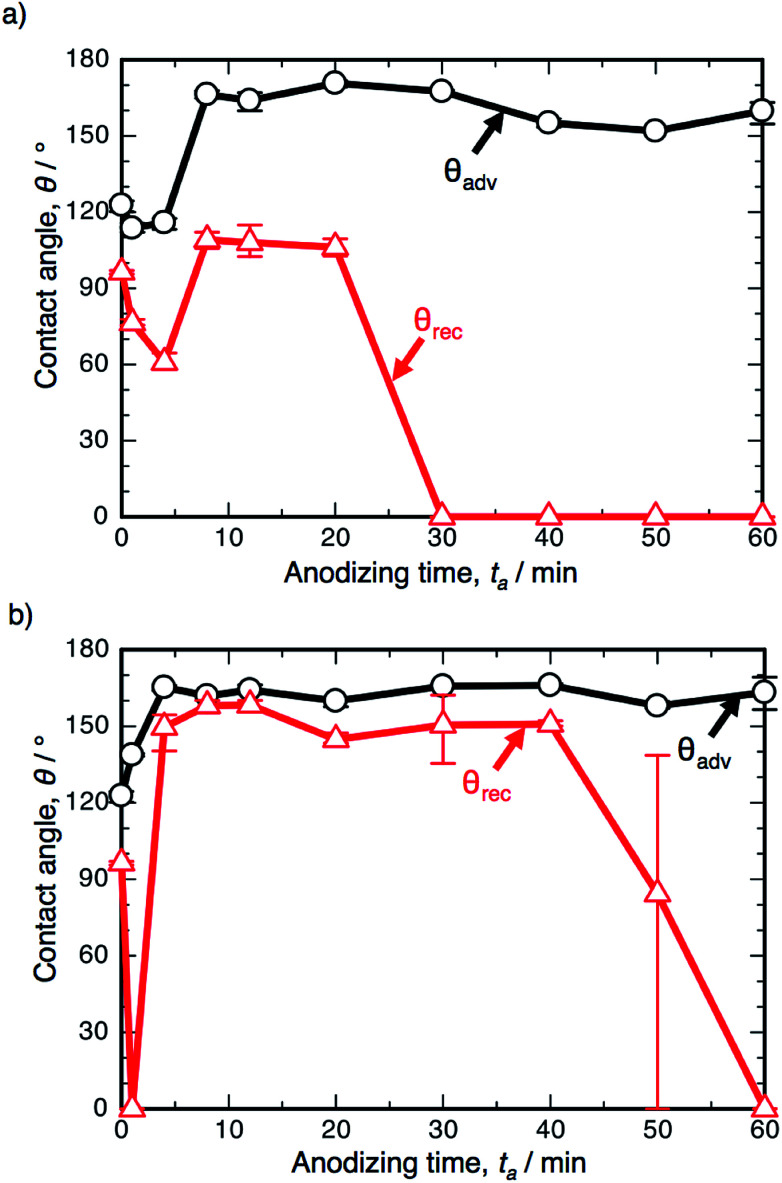
Changes in the advancing contact angle, *θ*_adv_, and receding contact angle, *θ*_rec_, on the anodized specimen with anodizing time, *t*_a_. The specimens were anodized in concentrated pyrophosphoric acid solution at 293 K and (a) 25 V or (b) 75 V.

Fig. 6 shows the results of the WCA measurements on the SAM-modified aluminum specimens formed by PyAA at (a) 25 V and (b) 75 V. At the low voltage of 25 V (Fig. 6a), *θ*_adv_ increased rapidly to over 150° in the initial stage of PyAA, and this high value was maintained on the anodized surface for up to 60 min. The shape of the *θ*_adv_ curve is similar to that obtained at 50 V (Fig. 3), even though the surface slowly changed to show superhydrophobic behavior. The shape of the *θ*_rec_ curve is also similar to the results obtained at 50 V, but the variation is smaller; *θ*_rec_ decreased slightly to 60° after 4 min, slightly increased to 109° after 8 min, and then rapidly decreased to 0° after more than 30 min.

When the anodizing voltage was increased to 75 V (Fig. 6b), *θ*_adv_ rapidly increased to over 150° within 4 min, and this high value was maintained upon further PyAA. Therefore, the surface formed by high-voltage anodizing rapidly changed to show superhydrophobic behavior. In addition, *θ*_rec_ changed drastically with anodizing time; *θ*_rec_ rapidly decreased to 0° upon anodizing for 1 min, rapidly increased to over 150° after 4 min, maintained its value for up to 40 min, and then gradually decreased to 0° upon further PyAA. Such changes are more drastic than those obtained at 50 V. Notably, the highest hysteresis measured at 163.2° and the lowest hysteresis measured at 3.8° were obtained by PyAA at 75 V.

The WCA measurements and the corresponding nanostructures formed by PyAA can be summarized as follows. As the alumina nanofibers were formed by PyAA, the surface exhibits superhydrophobic behavior with *θ*_adv_ = 150°. This superhydrophobicity is maintained by the subsequent bundle and weak structures formed by further PyAA. In contrast, the change in *θ*_rec_ is much more complex; *θ*_rec_ decreases as the porous oxide is formed on the surface, increases as the alumina nanofibers and the bundle structures are formed, and decreases once again as the weak nanofiber structures are formed. As a result, the contact angle hysteresis increases, decreases, and then increases once again with increasing anodizing time.

**Fig. 7:**
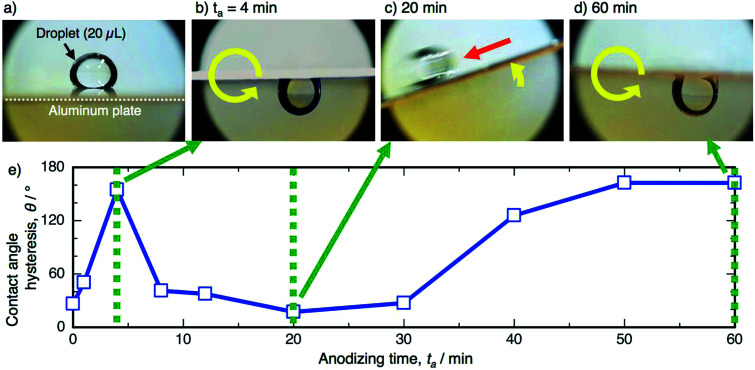
(a) Photographs of the 20 μL water droplet on the specimen anodized at 50 V. The specimens were rotated counterclockwise at 5° s^−1^ after the droplet was placed on the surface anodized for (b) 4 min, (c) 20 min, and (d) 60 min. (e) Change in the corresponding contact angle hysteresis with anodizing time.

The contact angle hysteresis corresponds to the sliding behavior of the water droplet on the surface. A direct observation of the sliding behavior was recorded by a digital microscope, and still images and the corresponding contact angle hysteresis curve are shown in Fig. 7. Here, a water droplet (20 μL) was placed on the horizontal specimen anodized at 50 V (Fig. 7a), and then, the specimens were rotated counterclockwise at a rate of 5° s^−1^. The water droplet strongly adhered on the specimen anodized for 4 min after the 180° rotation, and a highly sticky aluminum surface was obtained, as shown by the high hysteresis of 154.7° (Fig. 7b). As the contact angle hysteresis decreased to 17.3° after PyAA for 20 min, the water droplet easily slid to the left side upon tilting the specimen (Fig. 7c). As the hysteresis increased once again by further anodizing for 60 min, the droplet strongly adhered on the surface after the 180° rotation (Fig. 7d). Consequently, sticky aluminum surfaces could be obtained *via* short- and long-term anodizing processes, and slippery surfaces could also be obtained *via* the mid-term anodizing process.

**Fig. 8:**
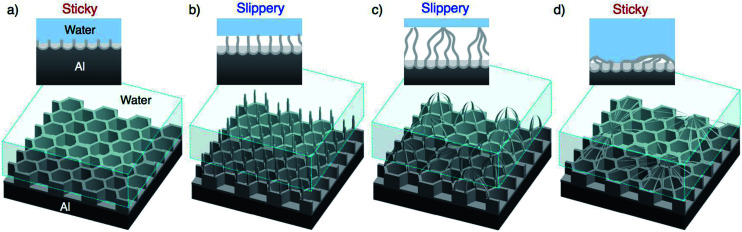
Schematic illustration of the growth behavior of the anodic alumina nanofibers formed by PyAA and the corresponding adhesion interactions between the water droplet and the nanostructured aluminum surface.

Fig. 8 shows schematic illustrations of the morphological variations and the expected results for a water droplet placed on the surface. The magnitude of the adhesive force of the water droplet increases in the order of:^56–58^Area-contact > point-contact

The porous oxide film formed by short-term PyAA has a continuous alumina area, and this area-contact surface may generate stronger surface adhesion than the point contact. Therefore, the water droplet is supported by the continuous area, and the surface exhibits sticky behavior (Fig. 8a). As the anodizing time increased, numerous independent alumina nanofibers and pyramidal bundle structures were formed on the surface (Fig. 8b and c). The water droplet is supported by the points of the alumina nanostructures, and this point-contact with the lowest contact area causes the highly slippery behavior (Cassie–Baxter state).^33,40^ Finally, as weak nanofiber structures were formed by excess PyAA, the surface once again exhibits sticky behavior due to its area-contact surface (Fig. 8d). Here, the water droplet is supported by the area-contact surface of the weak nanofiber structure (Cassie-impregnating wetting state).^33,40^ However, further investigations are required in order to develop a deeper understanding of these unique behaviors.

**Fig. 9:**
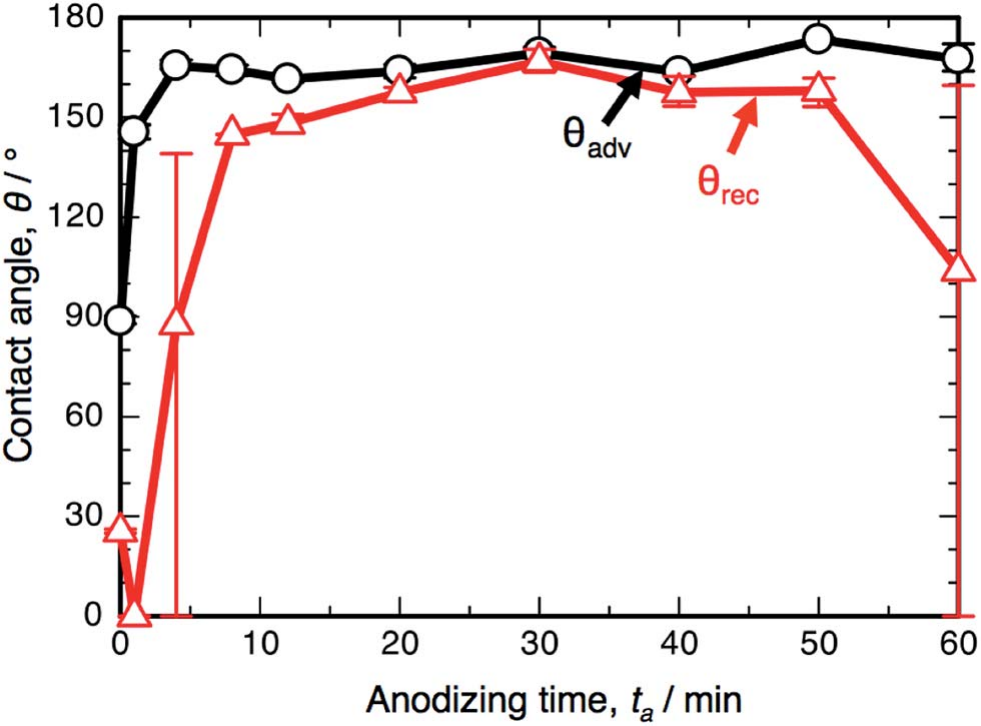
Changes in the advancing contact angle, *θ*_adv_, and receding contact angle, *θ*_rec_, on the anodized specimen with anodizing time, *t*_a_. The specimens were prepared by two distinct anodizing processes at 50 V using oxalic acid and pyrophosphoric acid for the formation of ordered alumina nanofibers.

Typical PyAA leads to the formation of disordered alumina nanofibers on the aluminum surface, and the nanostructure has many defects, such as doubled-nanofibers and non-uniform shapes.^21,22^ The sticky and slippery behaviors will be improved by the formation of ordered alumina nanofibers due to the complete point-contact. We previously reported that ordered alumina nanofibers were fabricated *via* two distinct anodizing processes.^27^ In this technique, an ordered aluminum dimple array was fabricated by typical OAA and porous alumina dissolution, and then, anodic alumina nanofibers were grown at the six apexes of the ordered dimples through PyAA at the same applied voltage. As a result, highly ordered alumina nanofibers without any defects were fabricated on the surface. Fig. 9 shows the WCA measurements on the SAM-modified aluminum specimens formed by the two distinct anodizing processes at 50 V. Compared to the surface obtained by one-step PyAA at 50 V (Fig. 3), (a) the *θ*_adv_ values were slightly higher and (b) the contact angle hysteresis was smaller after PyAA for 8–50 min. Therefore, the water droplet easily slid to the left side after slightly tilting the specimen at an angle of less than 2°, and a highly slippery aluminum surface was successfully obtained by the two distinct anodizing processes.

**Fig. 10:**
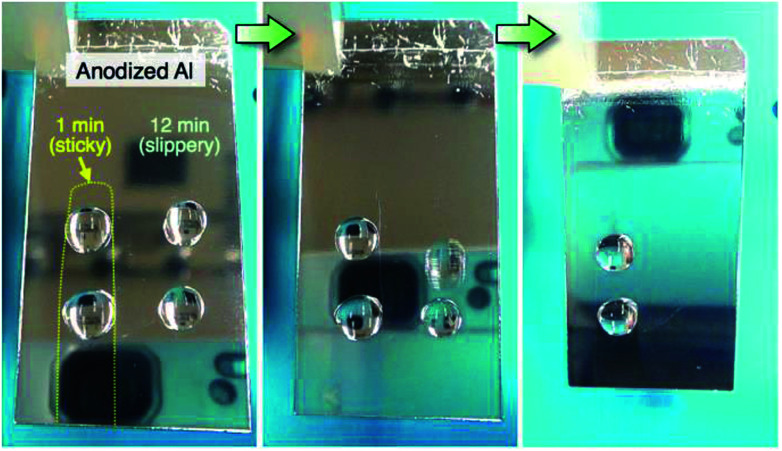
Water-sliding behavior of the aluminum surface fabricated *via* the selective anodizing method at 293 K and 75 V for 1 min (sticky surface) and 12 min (slippery surface).

Finally, we demonstrate the fabrication of a superhydrophobic aluminum surface showing both sticky and slippery properties through the selective anodizing method. In this technique, the electropolished aluminum specimens were selectively covered with a non-electroconductive nitrocellulose layer, and the exposed aluminum surface without the nitrocellulose layer was anodized in a pyrophosphoric acid solution at 293 K and 75 V for 11 min. The anodized specimens were immersed in acetone to dissolve the nitrocellulose layer and then anodized once again under the same operating conditions for 1 min. Consequently, two different anodized regions of the sticky surface (anodizing time: 1 min) and the slippery surface (total anodizing time: 12 min) were fabricated on the aluminum substrate. The water-sliding behavior of the anodized specimen is shown in Fig. 10 (a movie is also available in the ESI†). Here, two water droplets (20 μL) were placed on the surfaces anodized for 1 min and 12 min, and then, the specimen was slowly tilted to the front. The reflection of the digital camera was observed on the aluminum due to the highly mirror-finished surface formed by anodizing. The water droplet easily slid from the slippery surface anodized for 12 min, even though the droplet strongly adhered to the sticky surface anodized for 1 min. Such a superhydrophobic aluminum surface with the coexisting sticky and slippery properties could be easily fabricated *via* the selective anodizing method.

## Conclusions

We investigated the WCAs of nanofiber-covered aluminum surfaces fabricated by anodizing in pyrophosphoric acid and the modification of fluorinated SAMs. We successfully fabricated highly slippery and sticky superhydrophobic aluminum surfaces. The following conclusions were reached in our investigations.

(1) Anodizing aluminum in pyrophosphoric acid solution results in the growth of anodic alumina nanofibers through barrier and porous oxide formation. The nanofibers are bundled as the anodizing time increases, and the bundle structures grow during long-term anodizing. Finally, weak nanofiber structures are obtained on the surface by excess anodizing due to chemical dissolution of anodic alumina. The growth rate, density, and morphology of the alumina nanofibers depend strongly on the anodizing voltage.

(2) The contact angle hysteresis changes drastically with anodizing time and the corresponding morphology of the alumina nanofibers. The hysteresis increases with the formation of porous oxide, decreases for the nanofibers and bundle structures, and then increases once again for the weak nanostructures. Therefore, the adhesion interaction between the water droplet and the surface also drastically changes with anodizing time to sticky, slippery, and sticky. Higher sticky and slippery aluminum surfaces can be obtained by anodizing at higher voltages.

(3) The slippery behavior exhibited on the aluminum surfaces can be further improved by the use of two distinct anodizing processes through the formation of an aluminum dimple array and subsequent alumina nanofibers.

(4) A superhydrophobic aluminum surface with the coexisting sticky and slippery properties can be fabricated by the selective anodizing method.

## Conflicts of interest

There are no conflicts to declare.

## Supplementary Material

RA-008-C8RA07712F-s001

## References

[cit1] Gabe D. R. (2002). Met. Finish..

[cit2] Kuroda K., Okido M. (2018). J. Biomater. Nanobiotechnol..

[cit3] Moon S., Jeong Y. (2009). Corros. Sci..

[cit4] Kikuchi T., Takenaga A., Natsui S., Suzuki R. O. (2017). Surf. Coat. Technol..

[cit5] Li X., Nie X., Wang L., Northwood D. O. (2005). Surf. Coat. Technol..

[cit6] Moon S., Pyun S. (1998). J. Solid State Electrochem..

[cit7] Choi Y.-I., Salman S., Kuroda K., Okido M. (2013). Electrochim. Acta.

[cit8] Capelossi V. R., Poelman M., Recloux I., Hernandez R. P. B., de Melo H. G., Olivier M. G. (2014). Electrochim. Acta.

[cit9] Zheng M., Sakairi M., Jha H. (2012). Corros. Sci..

[cit10] Li J., Papadopoulos C., Xu J. M., Moskovits M. (1999). Appl. Phys. Lett..

[cit11] Lee W., Joon P. S. (2014). Chem. Rev..

[cit12] Yanagishita T., Kondo T., Masuda H. (2018). J. Vac. Sci. Technol., B: Nanotechnol. Microelectron.: Mater., Process., Meas., Phenom..

[cit13] Jha H., Kikuchi T., Sakairi M., Takahashi H. (2008). Nanotechnology.

[cit14] Lee W., Schwirn K., Steinhart M., Pippel E., Scholz R., Gösele U. (2008). Nat. Nanotechnol..

[cit15] Zaraska L., Sulka G. D., Jaskuła M. (2010). Surf. Coat. Technol..

[cit16] Li A. P., Müller F., Birner A., Nielsch K., Gösele U. (1999). J. Vac. Sci. Technol., A.

[cit17] Kikuchi T., Nakajima D., Nishinaga O., Natsui S., Suzuki R. O. (2015). Curr. Nanosci..

[cit18] Zaraska L., Jaskuła M., Sulka G. D. (2016). Mater. Lett..

[cit19] Nakajima D., Kikuchi T., Natsui S., Suzuki R. O. (2014). Appl. Surf. Sci..

[cit20] Akiya S., Kikuchi T., Natsui S., Suzuki R. O. (2017). Appl. Surf. Sci..

[cit21] Kikuchi T., Nishinaga O., Nakajima D., Kawashima J., Natsui S., Sakaguchi N., Suzuki R. O. (2014). Sci. Rep..

[cit22] Nakajima D., Kikuchi T., Natsui S., Sakaguchi N., Suzuki R. O. (2015). Appl. Surf. Sci..

[cit23] Meier L. A., Alvarez A. E., Salinas D. R., del Barrio M. C. (2012). Mater. Lett..

[cit24] Zhao N.-Q., Jiang X.-X., Shi C.-S., Li J.-J., Zhao Z.-G., Du X.-W. (2007). J. Mater. Sci..

[cit25] Altomare M., Pfoch O., Tighineanu A., Kirchgeorg R., Lee K., Selli E., Schmuki P. (2015). J. Am. Chem. Soc..

[cit26] Nakajima D., Kikuchi T., Natsui S., Suzuki R. O. (2018). Appl. Surf. Sci..

[cit27] Nakajima D., Kikuchi T., Natsui S., Suzuki R. O. (2015). ECS Electrochem. Lett..

[cit28] Kondo R., Nakajima D., Kikuchi T., Natsui S., Suzuki R. O. (2017). J. Alloys Compd..

[cit29] Rong P., Yang W., Fu L., Zhu J., Li D., Zhou L. (2017). Mater. Res. Express.

[cit30] Nakajima D., Kikuchi T., Natsui S., Suzuki R. O. (2016). Appl. Surf. Sci..

[cit31] Otten A., Herminghaus S. (2004). Langmuir.

[cit32] Barthlott W., Neinhuis C. (1997). Planta.

[cit33] Feng L., Zhang Y., Xi J., Zhu Y., Wang N., Xia F., Jiang L. (2008). Langmuir.

[cit34] Lomga J., Varshney P., Nanda D., Satapathy M., Mohapatra S. S., Kumar A. (2017). J. Alloys Compd..

[cit35] Cao W.-T., Liu Y.-J., Ma M.-G., Zhu J.-F. (2017). Colloids Surf., A.

[cit36] Yao X., Song Y., Jiang L. (2011). Adv. Mater..

[cit37] Wang F., Liang C., Yang M., Fan C., Zhang X. (2015). Appl. Therm. Eng..

[cit38] Rungraeng N., Cho Y.-C., Yoon S. H., Jun S. (2012). J. Food Eng..

[cit39] Winkleman A., Gotesman G., Yoffe A., Naaman R. (2008). Nano Lett..

[cit40] Ebert D., Bhushan B. (2012). J. Colloid Interface Sci..

[cit41] Hong X., Gao X., Jiang L. (2007). J. Am. Chem. Soc..

[cit42] Schmitt M. and Heib F., in Advances in Contact Angle, Wettability and Adhesion 3, ed. K. L. Mittal, Wiley-Scrivener, Beverly, MA, 2018, section 1, pp. 1–57

[cit43] Marmur A. , in Contact Angle Wettability and Adhesion 6, ed. K. L. Mittal, CRC Press, Boca Raton, FL, 2009, pp. 3–18

[cit44] Schmitt M., Heib F. (2013). J. Chem. Phys..

[cit45] Schmitt M., Hempelmann R., Ingebrandt S., Munief W., Durneata D., Groβ K., Heib F. (2014). Int. J. Adhes. Adhes..

[cit46] Schmitt M., Grub J., Heib F. (2015). J. Colloid Interface Sci..

[cit47] Ma W., Wu H., Higaki Y., Otsuka H., Takahara A. (2012). Chem. Commun..

[cit48] Vengatesh P., Kulandainathan M. A. (2015). ACS Appl. Mater. Interfaces.

[cit49] Ulman A. (1996). Chem. Rev..

[cit50] Nakayama K., Tsuji E., Aoki Y., Habazaki H. (2014). RSC Adv..

[cit51] Marmur A., Volpe C. D., Siboni S., Amirfazli A., Drelich J. W. (2017). Surf. Innovations.

[cit52] Gao L., McCarthy T. J. (2006). Langmuir.

[cit53] Chibowski E. (2003). Adv. Colloid Interface Sci..

[cit54] Thompson G. E. (1997). Thin Solid Films.

[cit55] Sulka G. D., Stępniowski W. J. (2009). Electrochim. Acta.

[cit56] Lai Y., Gao X., Zhuang H., Huang J., Lin C., Jiang L. (2009). Adv. Mater..

[cit57] Öner D., McCarthy T. J. (2000). Langmuir.

[cit58] Chen W., Fadeev A. Y., Hsieh M. C., Öner D., Youngblood J., McCarthy T. J. (1999). Langmuir.

